# *Frutexites*-like structures formed by iron oxidizing biofilms in the continental subsurface (Äspö Hard Rock Laboratory, Sweden)

**DOI:** 10.1371/journal.pone.0177542

**Published:** 2017-05-19

**Authors:** Christine Heim, Nadia-Valérie Quéric, Danny Ionescu, Nadine Schäfer, Joachim Reitner

**Affiliations:** 1 Department of Geobiology, Geoscience Centre, University of Göttingen, Göttingen, Germany; 2 Leibnitz Institute for Freshwater Ecology and Inland Fisheries, IGB, Experimental Limnology, Stechlin, Germany; The University of Akron, UNITED STATES

## Abstract

Stromatolitic iron-rich structures have been reported from many ancient environments and are often described as *Frutexites*, a cryptic microfossil. Although microbial formation of such structures is likely, a clear relation to a microbial precursor is lacking so far. Here we report recent iron oxidizing biofilms which resemble the ancient *Frutexites* structures. The living *Frutexites*-like biofilms were sampled at 160 m depth in the Äspö Hard Rock Laboratory in Sweden. Investigations using microscopy, 454 pyrosequencing, FISH, Raman spectroscopy, biomarker and trace element analysis allowed a detailed view of the structural components of the mineralized biofilm. The most abundant bacterial groups were involved in nitrogen and iron cycling. Furthermore, Archaea are widely distributed in the *Frutexites*-like biofilm, even though their functional role remains unclear. Biomarker analysis revealed abundant sterols in the biofilm most likely from algal and fungal origins. Our results indicate that the *Frutexites*-like biofilm was built up by a complex microbial community. The functional role of each community member in the formation of the dendritic structures, as well as their potential relation to fossil *Frutexites* remains under investigation.

## Introduction

**Fig 1:**
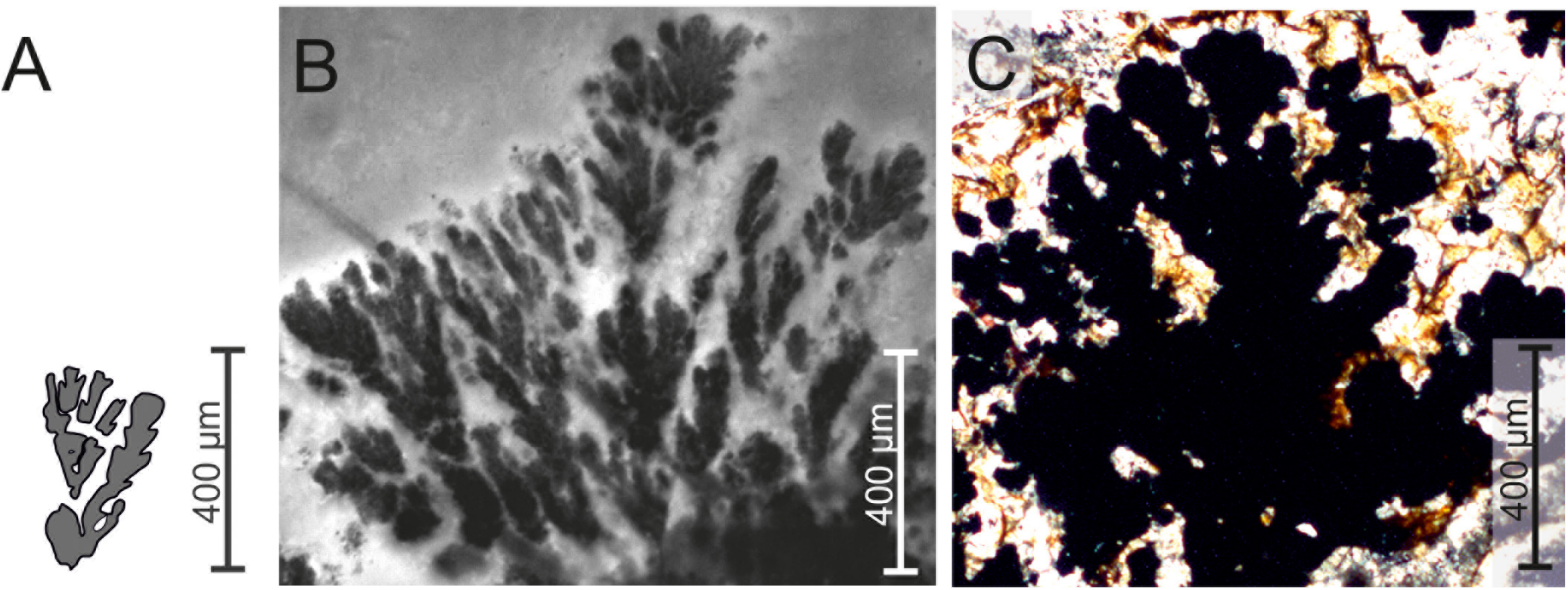
Comparison of the dendritic *Frutexites* structures modified after Maslov (18; A); recent forms from the Äspö HRL described in this paper (B) and fossil *Frutexites* from mineralized fractures of the “sole dolomite” at the base of the Naukluft Nappe Complex in southern Namibia [14].

Laminations and dendrites are common morphological features that allow us to correlate to Precambrian stromatolites to recent analogues formed by calcifying mineralizing microbes [e.g. 1, 2, 3, 4, 5, 6]. Other biomorphs observed in the geological record show similar characteristic shapes, but their formation processes are currently unclear. In numerous paleo-environmental studies, Fe-and Mn-rich stromatolitic and dendritic structures have often been described as *Frutexites*. In the fossil record, *Frutexites* occur in rock samples from different environments such as marine stromatolites, microbial limestones, hardgrounds, cavities, cracks, veins, and Neptunian dikes [e.g. 7, 8, 9, 10, 11, 12, 13, 14, 15, 16]. Major occurrences are described from rock cavities and fractures, mainly growing upward, but also radially to the nucleating surface [17].The genus *Frutexites* with five different species was first described by Maslow [18]. The identification and description of the fossil *Frutexites* in the literature is always based on the following character: dendritic structures mainly composed of iron and/or manganese oxides; several tens to hundreds of micrometres in height (Fig 1A; see [14] for an overview). However, clear evidence for the microbial biomineralization of *Frutexites* structures is lacking to date. With a recent analogue it should be possible to determine if and how microorganisms contribute to the formation of *Frutexites*.

Dendritic structures were observed in living iron oxide precipitating biofilms growing on rock surfaces in the Äspö Hard Rock Laboratory (HRL). The dendrites of the iron oxide biofilm strongly resemble the dendritic pattern of fossil *Frutexites* structures (Fig 1). In this study, we present a detailed, high resolution visualization of the morphological structures in the *Frutexites*-like biofilm. Furthermore, we aimed to identify the microbial community in the biofilm and their potential contribution to the dendrite formation. Biomarker and trace element analysis complement the data set and offer a link for biosignature investigations in fossil *Frutexite*s.

## Material & methods

### Sampling site

Samples for this study were taken from site NASA 1127B in the Äspö Hard Rock Laboratory (HRL) north of Oskarshamn, Sweden. The Äspö HRL is a tunnel excavated beneath the island of Äspö and is operated by SKB (Svensk Kärnbränsle Hantering AB which is the Swedish Nuclear Fuel and Waste Management Company) as a testing site for the long-term deposition of high-level radioactive nuclear waste. Access to the sampling site within the Äspö HRL was provided by SKB. The host rock of the Äspö HRL mainly consists of 1.8-Ga-old granitic to quartz-monzodioritic rocks belonging to the Precambrian Transscandinavian Igneous Belt [19].

**Fig 2:**
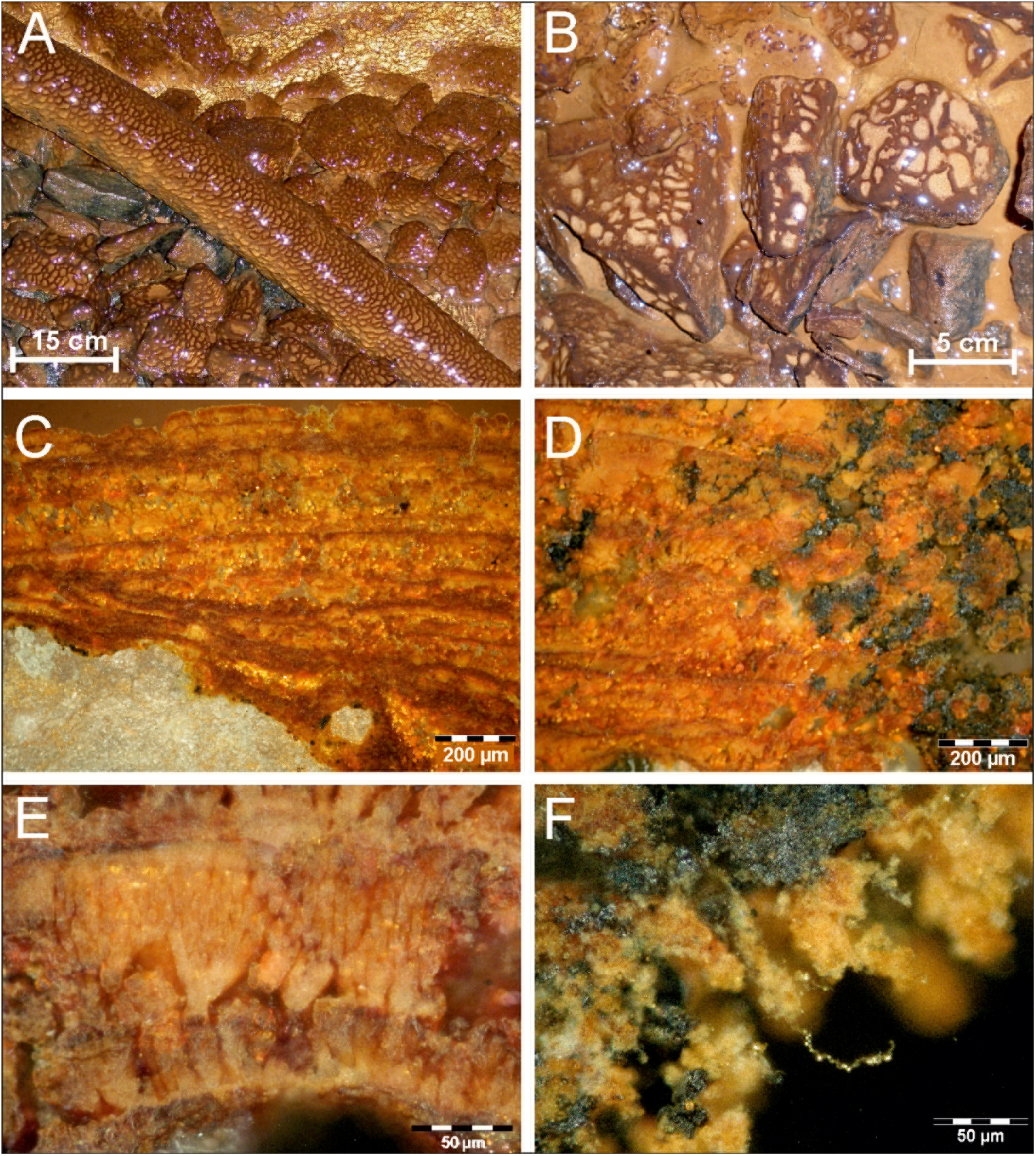
Iron oxidizing biofilms cover rocks and artificial tubes at sites NASA 1265A (A) and NASA 1127B (B) in the Äspö Hard Rock Laboratory at ca. 160 m depth. Cross sections of these *Frutexites*-like biofilms show an alternating dendritic and laminar growth, with varying contents of brown-red or blackish mineral phases (C; D). Enlarged dendritic mineralized structures within the laminae (E) and at the rim (F) of the biofilm.

The *Frutexites*-like biofilms form wherever iron-rich brackish water is dripping from the ceiling or trickling down the tunnel walls (Fig 2A and 2B). Most intense growth of the biofilms is observed at depths around 150 to 180 m below the surface. In this section of the tunnel, the groundwater originates from a brackish aquifer, which is influenced by Baltic Sea water [20, 21, 22].

### Histological methods

For TEM (transmission electron microscopy) and DOPE-FISH (double labeling of oligonucleotide probes for fluorescence *in situ* hybridization), samples were embedded in Technovit 7100 (GAM Kit: glycol methacrylate monomer, hardner I & II, Thermo Fisher Scientific, Braunschweig, Germany) as described in [23], serially sectioned into 2–3 μm thick slices by a rotary microtome and stored at 4°C until further processing. DOPE-FISH analyses on histological thin sections provide evidence of the distribution of prokaryotic cells close to the surface of the biofilm.

For FE-SEM (field emission scanning electron microscopy), samples were fixed in 2% glutaraldehyde immediately after sampling and stored at 4°C until analysis. Prior to measurement, the samples were dehydrated in ethanol dehydration series (concentration 15% to 99%). Final dehydration was performed by critical point drying (EM CPD030, Leica Microsystems, Heerbrugg, Switzerland) as well as chemically drying via HMDS (hexamethyldisilazane). Sample proportions were mounted on SEM sample holders and sputtered with Au-Pb (7.3 nm for 120 s). Samples were analysed using a field emission SEM (FE-SEM; LEO 1530 Gemini) combined with an INCA X-act EDX (Oxford Instruments).

For different microscopic investigations, samples were embedded in LR-White (for the preparation of hard part sections). For TEM the samples were embedded in methylacrylate and cut with an ultramicrotome using glass and diamond knives to gain 3 μm ultrathin sections. Technovit 7100 embedding was performed for cutting hard part thin sections stained with Alcian blue (0298-50G; VWR) to visualize acidic polysaccharides of membranes and extracellular polymeric substances (EPS).

### 454 pyrosequencing

DNA was extracted from 2 samples as described in [24]. The samples were sequenced using 454 pyrosequencing for bacterial diversity using the 28F and 519R primers [25]. Pyrosequencing was done by MrDNA laboratories (Shallowater, TX, USA) using a Roche 454 GS Titanium FLXC Genome Sequencer system. Briefly, Tag-encoded FLX amplicon pyrosequencing (bTEFAP) was carried out as previously described [26]. A 20 ng (1 ml) aliquot of each DNA sample was used for a 25 ml PCR reaction. A 30 cycle PCR using HotStarTaq Plus Master Mix Kit (Qiagen, USA) were used under the following conditions: 94°C for 3 min, followed by 30 cycles of 94°C for 30 sec; 55°C for 40 sec and 72°C for 1 min; and a final elongation step at 72°C for 5 min. Following PCR, all amplicon products from different samples were mixed in equal volumes and purified using Agencourt Ampure beads (Agencourt Bioscience Corporation, USA). A total of 17,598 sequences were obtained (in duplicate samples) and were processed as described in [27] using the SILVA NGS pipeline (https://www.arb-silva.de/ngs/). Briefly, the sequences were checked for quality (length, homopolymers and ambiguous bases) and aligned against the SILVA ref database [28]. Sequences with no alignment were removed while the rest were dereplicated and clustered (per sample) into operational taxonomic units (OTUs) using the CD-HIT EST software [29] at a similarity value of 100% and 98%, respectively. Reference sequences of each OTU ware assigned a taxonomic value using BLAST against the SILVA ref database (Version 111; [28]). Full OTU mapping of the sample was done by clustering the reference sequences of each OTU at 99% similarity.

The sequences were deposited at the Sequences Read Archive (SRA) under study accession number PRJEB4914 (http://www.ebi.ac.uk/ena/data/view/PRJEB4914).

### Biomarker analysis

The lyophilized biofilm (500 mg) was extracted with 20 mL of pre-distilled dichloromethane in Teflon-capped glass bottles (ultrasonication, 35 min, 40°C). The supernatant was decanted after centrifuging. Extraction was repeated three times. After evaporation of the combined extracts and re-dissolution in pure dichloromethane, the solvents were dried with N_2_. The total organic extract (TOE) was re-dissolved with n-hexane. An aliquot (25%) of the TOC was derivatized by adding 100 μL BSTFA/pyridine (40°C, 1.5 h). The collected supernatant was dried with N_2_, and then mobilized with 300μl n-hexane with deuterated eicosane (C_20_D_42_; 4 mg/l) as internal standard. 1 μL of the sample extract was analysed via on-column injection into a Varian CP-3800 GC/1200-quadrupole MS (70keV) equipped with a fused silica column (Phenomenex ZB-5; 30 m length, 0.32 μm inner diameter.; 0.25 μm film thickness). The GC oven was programmed from 80°C (held 3 min) to 325°C (held 40 min) at 6°C/min. He was used as carrier gas at 1.4 ml/min. Compounds were assigned by comparison with published mass spectral data.

### ICP-OES and ICP-MS analysis

Major cations and rare earth elements (REE-Y) were analyzed in two *Frutexites*-like biofilms. 4 ml of H_2_O_2_ (Carl Roth; 35%) and 2 ml of concentrated, distilled HNO_3_ (Merck, triple distilled in the GZG geochemistry lab) were added to 500 mg of the lyophilized sample. The resulting solutions of the mineral precipitates were diluted in 50 ml of deionized water (final concentration 4% HNO_3_). These solutions, two aquifer samples, and a reference sample (matrix matched calibration solution) containing all chemicals used, were spiked with internal Ge, Rh, In and Re standards and analyzed by ICP-OES (optical emission spectroscopy; Perkin Elmer Optima 3300 DV) and ICP-MS (inductive coupled plasma—mass spectrometry; Perkin Elmer SCIEX Elan DRCII). The values are mean values of triplicate measurements. Furthermore, laser ablation-ICP-MS (LA-ICP-MS) was conducted on several different spots on cross sections of two different *Frutexites*-like biofilms to better detect chemical variations in iron oxides that cannot be observed in the bulk extract. For a potential comparison with ancient analogs REE-Y data from each sample (LA-ICP-MS 1–5; aquifer and the dissolved biofilm) are normalized to PAAS (Post-Archaean average Australian sedimentary rock; [30]).

### Carbon-phase analyses and carbon, nitrogen and sulfur (CNS) measurements

Inorganic carbon (C_inorg_) and organic carbon (C_org_) were analyzed with a Leco RC 412 multiphase carbon analyzer. Carbon (C_tot_), total nitrogen (N_tot_) and sulfur (S_tot_) were analyzed using a CNS Elemental Analyzer (HEKAtech Euro EA). Duplicate measurements were performed routinely, and appropriate internal standards were used for the C_inorg_ (Leco 502–030), C_org_ (Leco 501–034), and CNS (2,5-Bis(5-tert-butyl-2-benzoxazolyl)thiophene, SA990752; atropine sulfate SA990753; IVA Analysentechnik) analyses.

### Raman spectroscopy

Raman spectra were recorded using a confocal Horiba Jobin-Yvon LabRam-HR 800 UV Raman spectrometer with attached Olympus BX41 microscope and an argon ion laser (Melles Griot IMA 106020B0S; 488nm) with a laser power of 20 mW at the laser exit (ca. 3–4 mW at the sample). The focal length of the spectrometer of 800 mm and the use of a 600 l/mm grating and a CCD detector with 1024 x 256 pixels yielded a spectral dispersion of ≤2 cm^-1^ per pixel. Using an Olympus MPlane 100x objective with a numerical aperture of 0.9 and closing the confocal hole to 100 μm resulted in a lateral resolution of ca. 1 μm and a depth resolution of ca. 5 μm. The acquisition time varied between 10 and 30 s for a spectral range of 100–4000 cm^-1^. For calibration of the spectrometer a silicon standard with a major band at 520.4 cm^-1^ was used. All spectra were recorded and processed using LabSpecTM version 5.19.17 (Horiba Jobin-Yvon, Villeneuve d’Ascq, France). The minerals were identified on the basis of the Horiba Jobin-Yvon database for minerals and reference spectra collected from mineral specimens of the Geoscience Museum of the Georg-August University Göttingen.

## Results

### Structure of the mineralized biofilm

**Fig 3:**
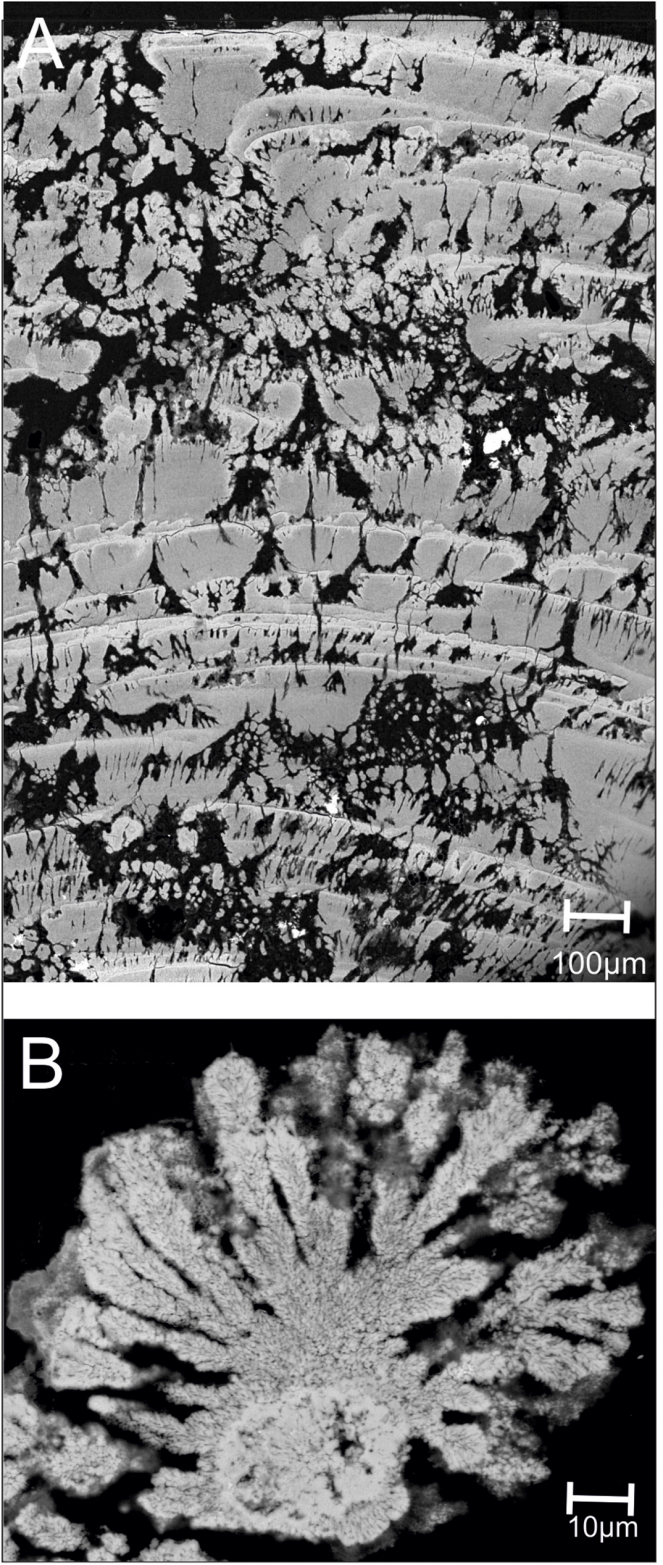
FE-SEM images showing cross sections of the iron oxidizing *Frutexites*-like biofilms (A; B). Brighter areas belong to dense mineral phases (A). Dendrites grow radially, mainly upwards from a nucleus.

The mineralized biofilms have a brown color, and show a net-like distribution with dark brown ridges and bright depressions on rocks or other surfaces (Fig 2A and 2B). Cross-sections of biofilms show a distinctive stromatolitic character in the mm to cm range, consisting of red-brown laminae with internal dendritic structures (Fig 2C) and, in places, intertwined black mineral aggregates (Fig 2D). A higher resolution shows the dendritic columns (Fig 2E) and different mineral aggregates also attached to organic filaments (Fig 2F). Such filamentous or net-like structures are mainly composed of extracellular polymeric substances (EPS; see below). Field emission scanning electron microscopy (FE-SEM) reveals brighter areas of higher mineral density within the laminated structure in cross section of the biofilms (Fig 3A). Dendritic iron oxides seem to grow almost radially from a nucleating core (Fig 3B).

**Fig 4:**
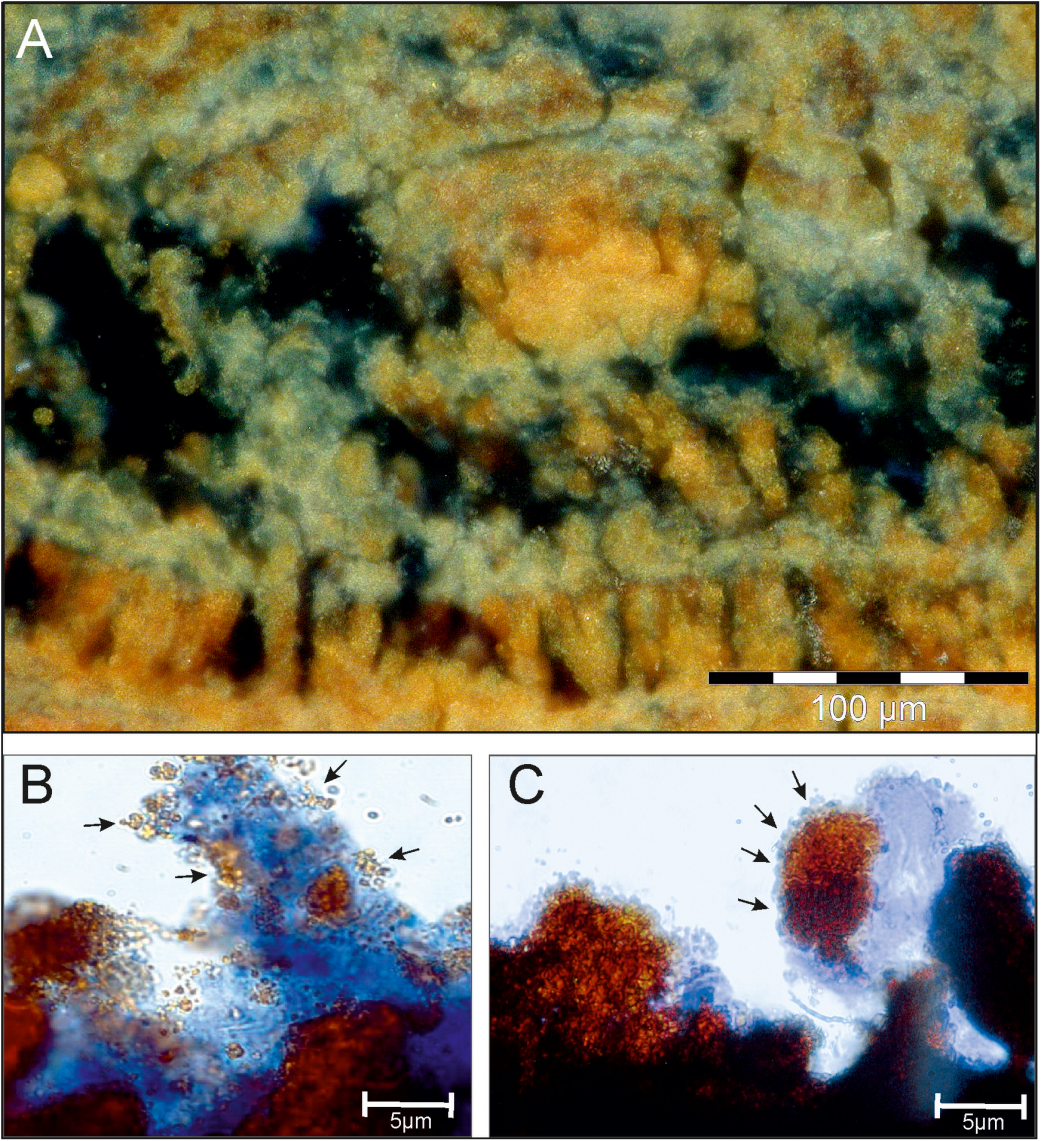
LR White embedded section stained with Alcian blue shows the EPS matrix in the mineralized biofilm. The EPS is distributed on the surface as well as in greater depths of the biofilm, surrounding the iron oxide layers and tips of the dendrites (A). Within the blue stained EPS matrix, mineral particles are entrapped (B) and form larger mineral aggregates (C), building up the characteristic dendrites.

Microscopical investigations of Alcian blue stained sections show the EPS around and within the mineral aggregates (Fig 4A). Almost every dendrite is covered in, or appears to be composed of permineralized EPS. Close-up-views show mineral particles of different sizes entrapped and accumulated within the EPS matrix (Fig 4B and 4C arrows).

### Microbial consortia and EPS structure

**Fig 5:**
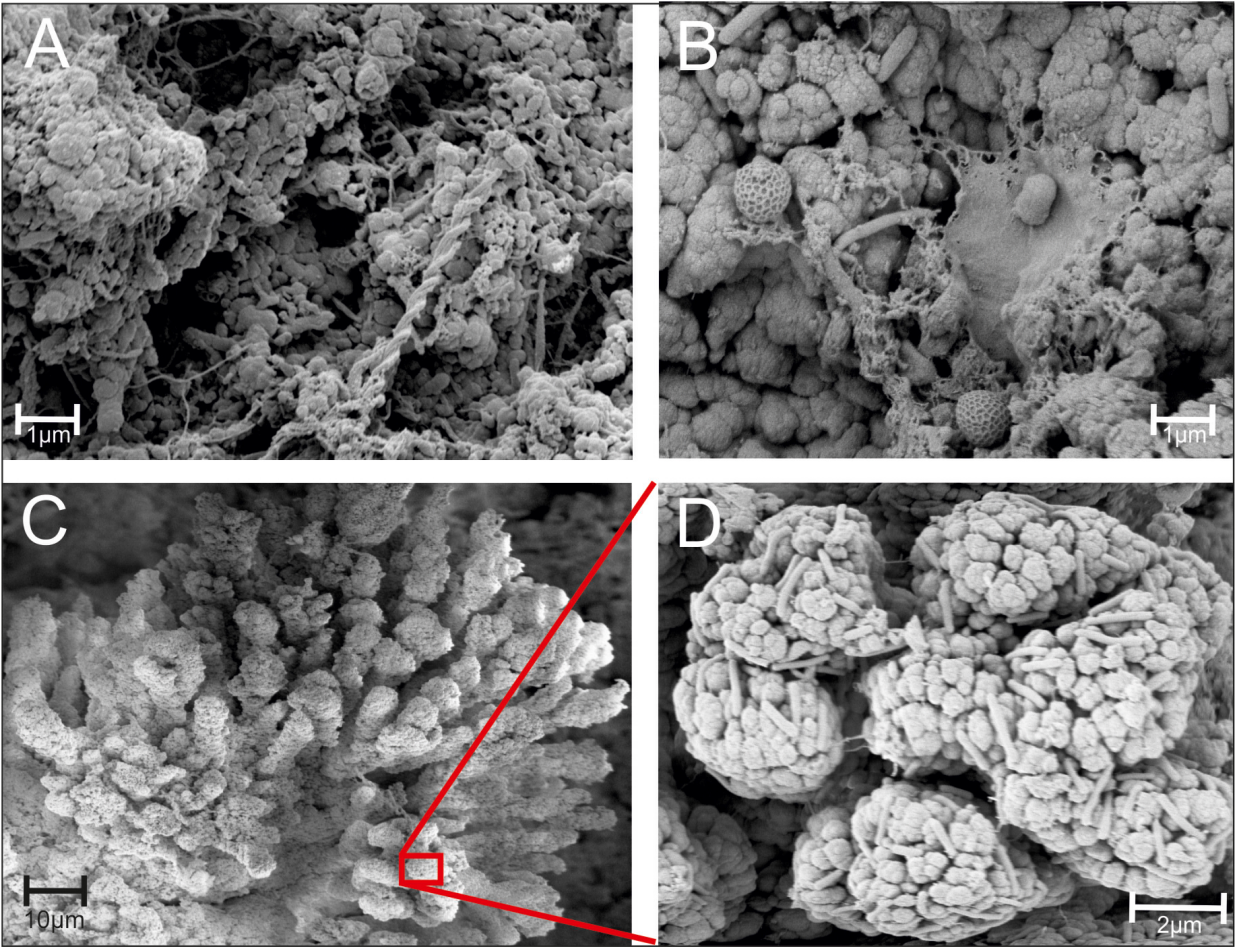
FEM images of a *Frutexites*-like biofilm. Various microbial EPS structures (A) and prokaryotic cells (B) are visible between the mineral precipitates. The dendrites (C) are covered with numerous rod-shaped microbial cells (D).

FEM investigations enable the visualization of the complex EPS fabrics and different cell morphologies within and on top of the mineral aggregates (Fig 5). Network-forming filaments, twisted stalks and agglomerates appear to be particularly abundant within cavities or depressions of the mineralized biofilm. A variety of morphologically distinct microbial structures were localized within the biofilm, including coccoid-, rod-shaped and filamentous microbes (Fig 5B). The mineralized dendrites are composed of roundish or seed like mineral grains and appear to be inhabited by abundant rod shaped microbes (Fig 5C and 5D).

**Fig 6:**
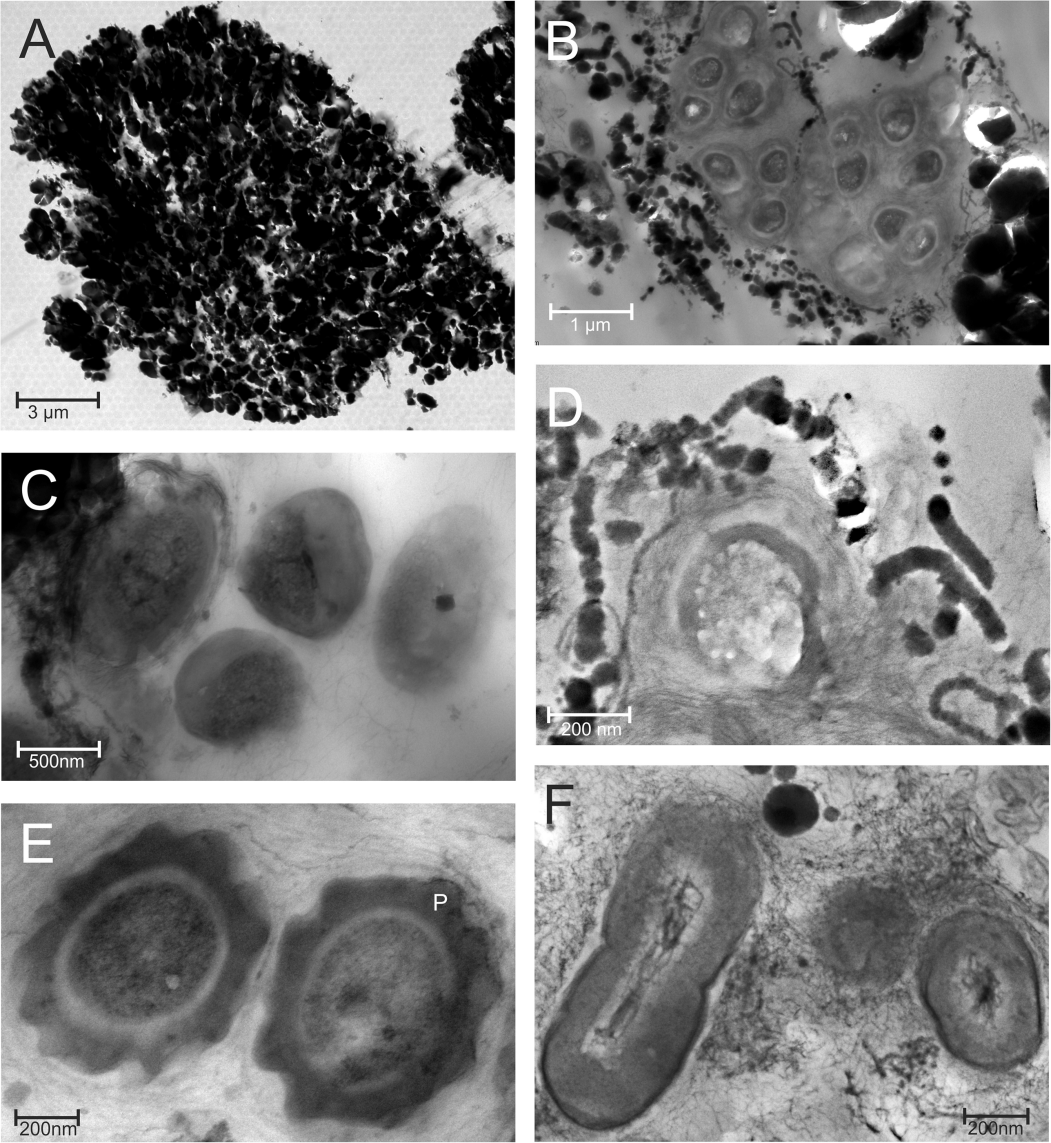
TEM micrographs of microbes detected in the *Frutexites*-like biofilm. The cross section of a dendritic structure shows a dense accumulation of mineral particles (A). In areas with less dense particles various microbial cells are observed (B-F). Most of the microbes are surrounded by a matrix composed of EPS and mineral particles, of either net-like (B, C, F) or laminated (D, E) structure. The dark, ca 50 nm wide square was interpreted as a magnetite produced within a magnetotactic bacterium (C). *Nitrotoga* cells were identified according to their characteristic shape and wide irregular periplasmic space (marked with P) (E). Dividing cells surrounded by a thick EPS matrix with a net like distribution of mineral particles (F).

Ultrathin sections from the central area of a biofilm revealed that these dendrites are built up from up to 1 μm sized mineral particles (Fig 6A). Microbial cells occur in densely packed EPS aggregates, surrounded by mineral particles and in close vicinity to dendrites (Fig 6B). In some microbial cells small dark cubic mineral particles were detected (Fig 6C). The shape of these particles, their occurrence and accumulation within the prokaryotic cell are characteristic traits of magnetotactic bacteria. The presence of magnetotactic bacteria *(Magnetospirillum magneticum)* was confirmed by sequencing analysis (see below). Chain-like mineral aggregates were detected around some bacterial cells (Fig 6D).

Furthermore, *Nitrotoga* bacterial cells with their characteristic irregular periplasmic space [31] were identified in the EPS matrix (Fig 6E) as well as in the DNA sequences (see below). Nearly all prokaryotic cells in the biofilm appear to be embedded in thin foliated EPS layers (Fig 6C; 6D and 6F). Although abundant mineral precipitates in the EPS matrix surround the microbial cells, they do not appear to hamper microbial growth and cell division (Fig 6F).

**Fig 7:**
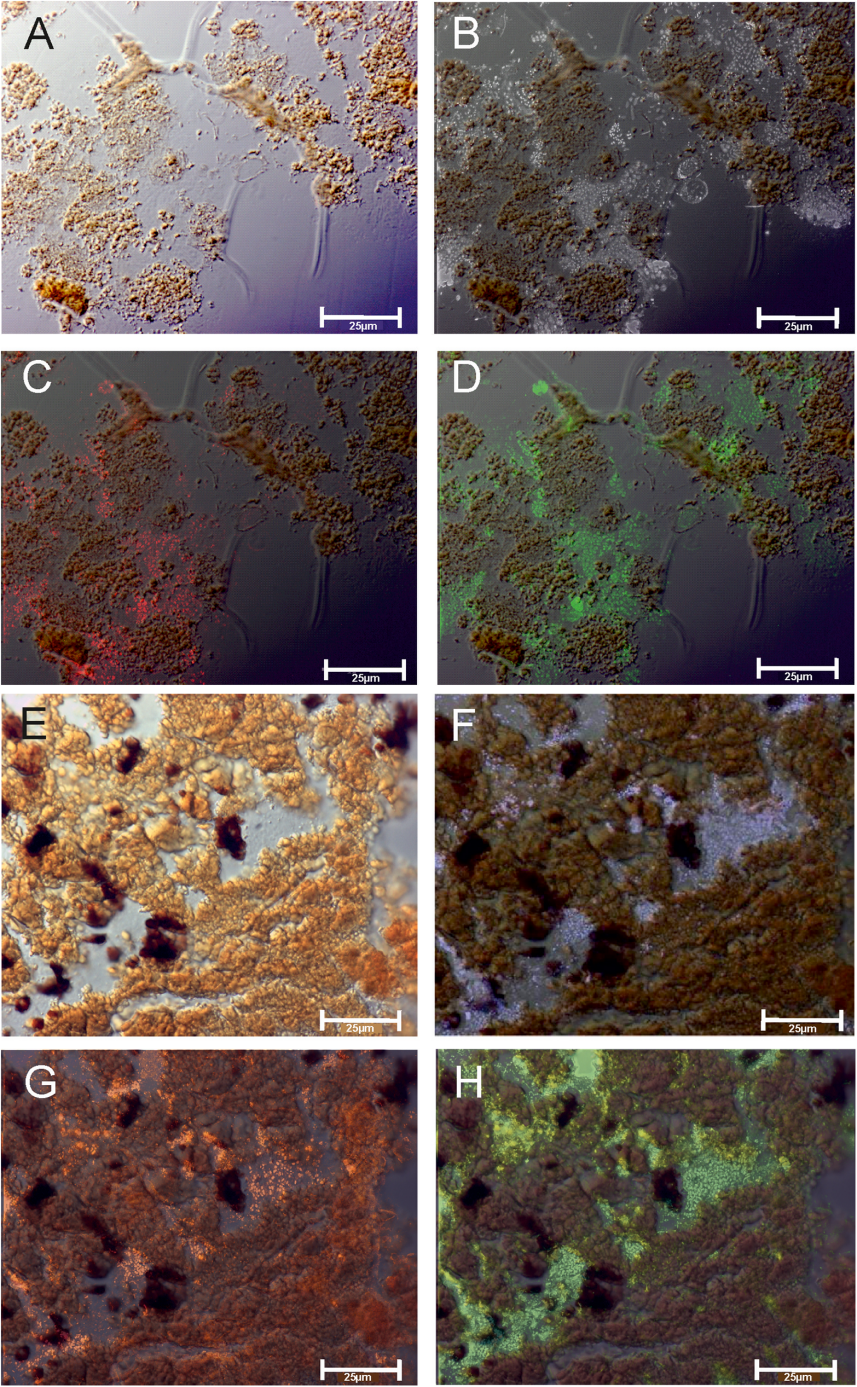
Transmitted light microscopy of an embedded *Frutexites* section at the top (A) and in the center of the biofilm (E). Epifluorescence microscopy on the DAPI stained section exhibits the distribution of prokaryotic cells (B, F). DOPE-FISH further distinguishes bacterial aggregates by probe EUB I-III-cy3 (C, G) and Archaea via probe Arch915-oregon green (D, H).

DOPE-FISH analyses revealed that bacteria are concentrated in distinct areas of the biofilm (Fig 7C and 7G red), whereas the Archaea are evenly abundant in the upper layers as well as in the deeper parts of the biofilm (Fig 7D and 7H; green).

**Fig 8:**
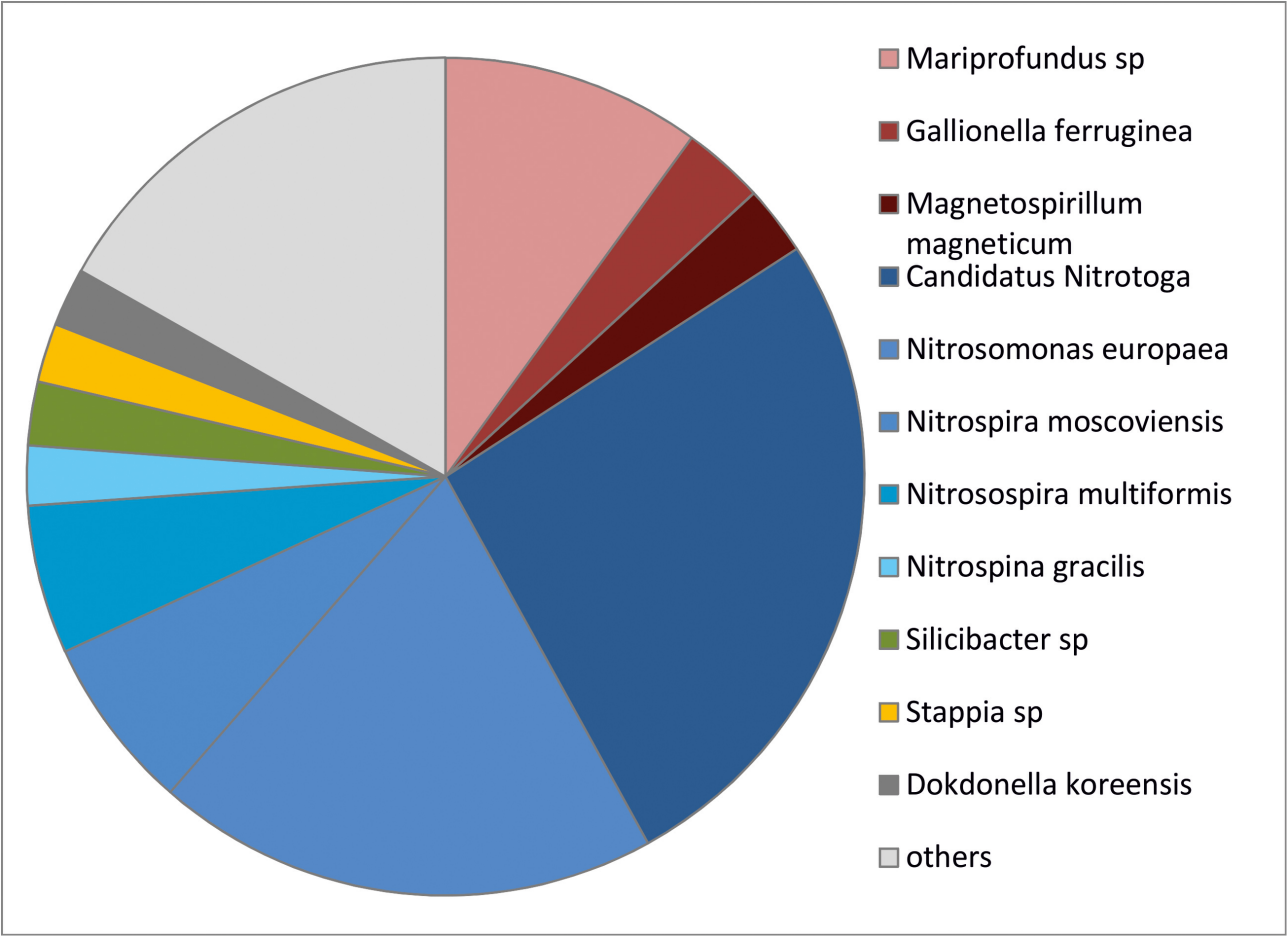
Sequence-based bacterial diversity of the *Frutexites*-like biofilm. The biofilm is dominated by nitrite and ammonia oxidizing bacteria (blue colors) and iron metabolizing bacteria (red colors). Both biofilms investigated show the same diversity pattern.

The bacterial community within the stromatolitic biofilm was elucidated by 454 pyro sequencing. Within the biofilm, almost 40% of the sequences determined were associated with bacteria involved in the nitrogen cycle (Fig 8). Ammonia- oxidizing bacteria (AOB) consisted of two different *Nitrosomonas* species, and nitrite oxidizing bacteria (NOB) comprised *Candiatus Nitrotoga*, *Nitrospira*, and *Nitrospina*. The second major group of organisms were iron oxidizing bacteria (FeOB), represented by *Mariprofundus sp*., *and Gallionella ferruginea*. The facultative anaerobic iron reducer *Magnetospirillum magneticum*, a magnetotactic bacterium is also present. This community composition indicates a close coupling between the iron and the nitrogen cycle.

### Lipid composition

**Fig 9:**
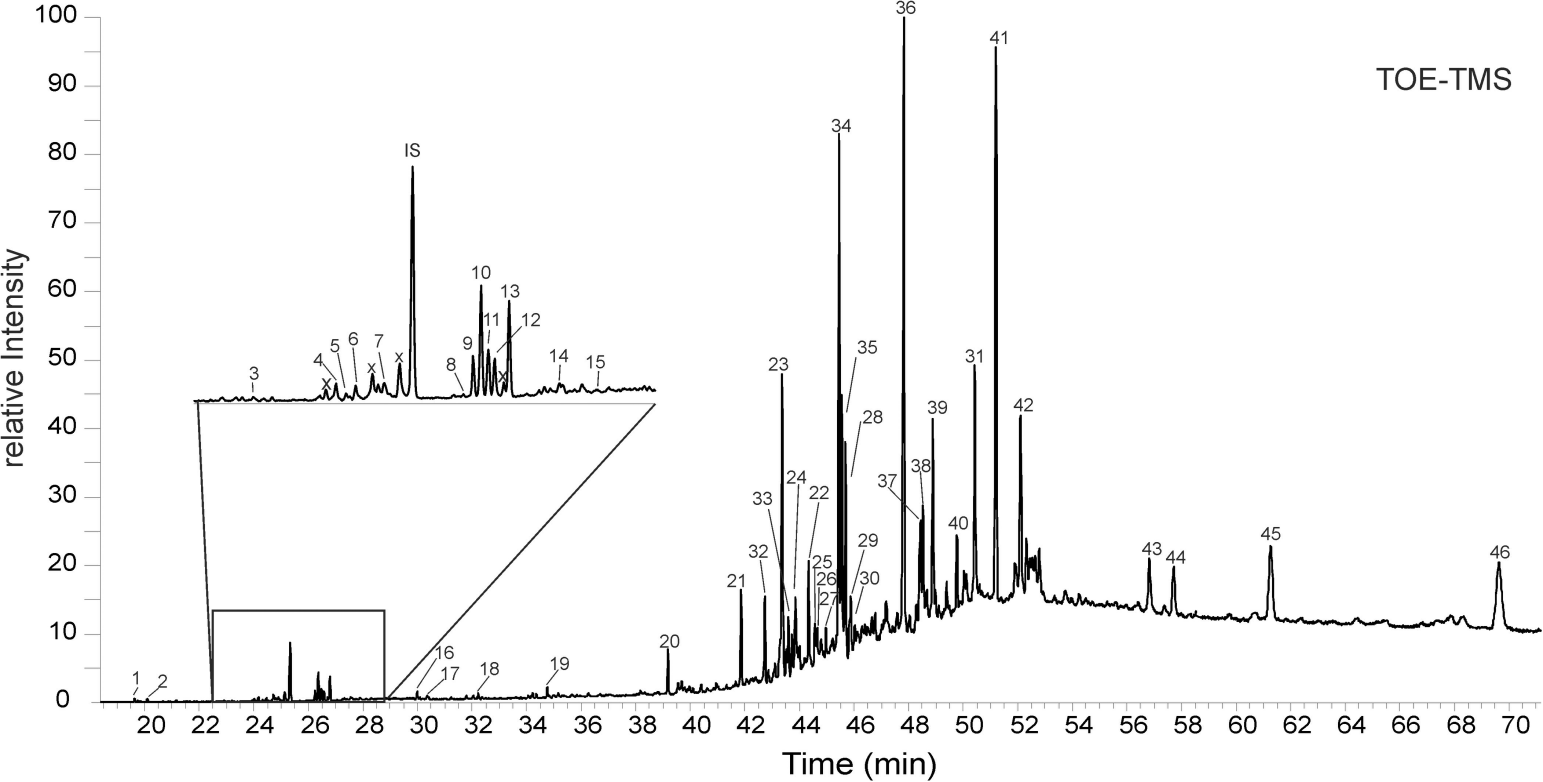
GC-MS chromatogram of the derivatized total organic extract from the *Frutexites*-like biofilm. The peak assignments are given in Table 1. Peaks labeled with “x” are non-derivatized fatty acids. IS = internal standard. See text for further details.

Compounds detected with GC/MS are shown in the chromatogram in Fig 9 and listed in Table 1. The total organic extract (TOE) of the *Frutexites*-like biofilm contains numerous organic compounds, mainly fatty acids (FA; 10%), sterols (21%) and hopanoids (69%). Fatty acids, range from C_14_ to C_20_, mainly with even carbon numbers. Among the FA C_16_ and C_18_ compounds are most abundant. Four different unsaturated C_16:1_ FA were observed and one C_18:1_ FA. The branched FAs, C_15_ iso (i) and anteiso (ai), C_16_i¸ 10methyl-C_16_ and C_17_i were detected. In the triterpenoid range, cholesterol (cholest-5-en-3β-ol), ergosta-5,22-dienol (24-methylcholesta-5,22-dien-3-β-ol), ergosta-5,7-dienol (24-methylcholesta-5,7-dien-3-β-ol), campesterol (24-methylcholest-5-en-3β-ol), sitosterol (24-ethylcholest-5-en-3β-ol) and sitostanol (24-ethylcholestan-3β-ol) were detected. Furthermore, partly functionalized hopanoids range from C_30_ to C_35_ were present, including diploptene (~9%), diplopterol (~11%), 17ß(H),21ß(H)-bishomohopanoic acid (~5%) 17ß(H),21ß(H)-bishomohopanone (~13%), 17ß(H),21ß(H)-bishomohopanol (C_32_-hopanol; ~5%), 17ß(H),21ß(H)-bacteriohopanetetrol (~6%). All compounds detected are given in Table 1.

**Table 1:** Biomarkers detected in the total organic extract of the *Frutexites*-like biofilm from Äspö tunnel.

Compound class	RT	Peak No.	MW	Formula	Compound	%
	19.62	1	168	C_16_H_30_O	Hexadecenal	0.05
	20.1	2			Unknown compound	0.06
Fatty acids	22.92	3	300	C_17_H_36_O_2_Si	C_14:0_ TMS	0.03
	24.18	4	314	C_18_H_38_O_2_Si	C_15:0_ i TMS	0.09
	24.34	5	314	C_18_H_38_O_2_Si	C_15:0_ ai TMS	0.04
	24.47	6	312	C_18_H_36_O_2_Si	C_15:1_ TMS	0.10
	24.91	7	314	C_18_H_38_O_2_Si	C_15:0_ TMS	0.08
	26.09	8	328	C_19_H_40_O_2_Si	C_16:0_ i TMS	0.02
	26.25	9	326	C_19_H_38_O_2_Si	C_16:1_ TMS	0.15
	26.37	10	326	C_19_H_38_O_2_Si	C_16:1_ TMS	0.43
	26.48	11	326	C_19_H_38_O_2_Si	C_16:1_ TMS	0.19
	26.58	12	326	C_19_H_38_O_2_Si	C_16:1_ TMS	0.16
	26.8	13	328	C_19_H_40_O_2_Si	C_16:0_	0.39
	27.61	14	342	C_20_H_42_O_2_Si	10MeC_16:0_ TMS	0.07
	27.95	15	342	C_20_H_42_O_2_Si	C_17:0_ i TMS	0.04
	29.99	16	354	C_21_H_42_O_2_Si	C_18:1_ TMS	0.16
	30.36	17	356	C_21_H_44_O_2_Si	C_18:0_ TMS	0.08
	32.21	18	370	C_22_H_46_O_2_Si	C_19:0_ TMS	0.14
	34.71	19	384	C_23_H_48_O_2_Si	C_20:0_ TMS	0.21
	39.18	20	410		Squalene	0.86
	41.85	21			Unknown compound	1.87
	44.34	22			Unknown compound	1.99
Sterols	43.36	23	458	C_30_H_54_OSi	Cholest-5-en-3β-ol TMS (cholesterol)	5.59
	43.86	24	470	C_31_H_54_OSi	24-Methylcholesta-5,22-dien-3-β-ol TMS (ergostan5,22-dienol)	1.88
	44.57	25	470	C_31_H_54_OSi	24-Methylcholesta-5,7-dien-3-β-ol TMS (ergostan5,7-dienol)	1.19
	44.65	26	472	C_33_H_54_OSi	24-Methylcholest-5-en-3β-ol TMS (campesterol)	1.04
	44.98	27	484	C_32_H_56_OSi	24-Ethylcholest-5, 22-en-3β-ol TMS (sitostadienol)	0.59
	45.69	28	486	C_32_H_58_OSi	24-Ethylcholest-5-en-3β-ol TMS (sitosterol)	4.01
	45.83	29	488	C_32_H_60_OSi	24-Ethylcholestan-3β-ol TMS (sitostanol)	0.61
	45.88	30	484	C_32_H_56_OSi	24-Ethylcholest-5-en-3β-ol TMS (sitosterol)	1.29
	50.42	31	504		Unknown steroid	5.22
Hopanoids	42.73	32	410	C_30_H_50_	Hop17(21)-ene	1.57
	43.72	33	384	C_28_H_48_	17β(H),18α(H),21β(H)-Bisnorhopane	1.02
	45.44	34	410	C_30_H_50_	Hop22(29)-ene (Diploptene)	9.34
	45.55	35	410	C_30_H_50_	Hop21(22)-ene	4.35
	47.82	36	428	C_30_H_52_O	Diplopterol	11.35
	48.44	37			Unknown hopanoid	2.38
	48.52	38			Unknown compound	2.12
	48.89	39			Unknown hopanoid	3.57
	49.77	40	440	C_32_H_56_	17ß(H),21ß(H)-Bishomohopane	1.89
	51.19	41	454	C_32_H_54_O	17ß(H),21ß(H)-Bishomohopanone	13.25
	52.09	42	528	C_34_H_60_O_2_Si	17ß(H),21ß(H)-Homohopanoic acid-TMS	5.14
	56.83	43			Unknown hopanoid	2.15
	57.71	44			Unknown hopanoid	2.12
	61.27	45	616	C_38_H_72_O_2_Si_2_	17ß(H),21ß(H)-Bishomohopandienol TMS	4.97
	69.65	46	834	C_47_H_94_O_4_Si_4_	17ß(H),21ß(H)-Bacteriohopanetetrol TMS	6.16
					Sum	100.00

### Mineralogy and chemical composition

**Fig 10:**
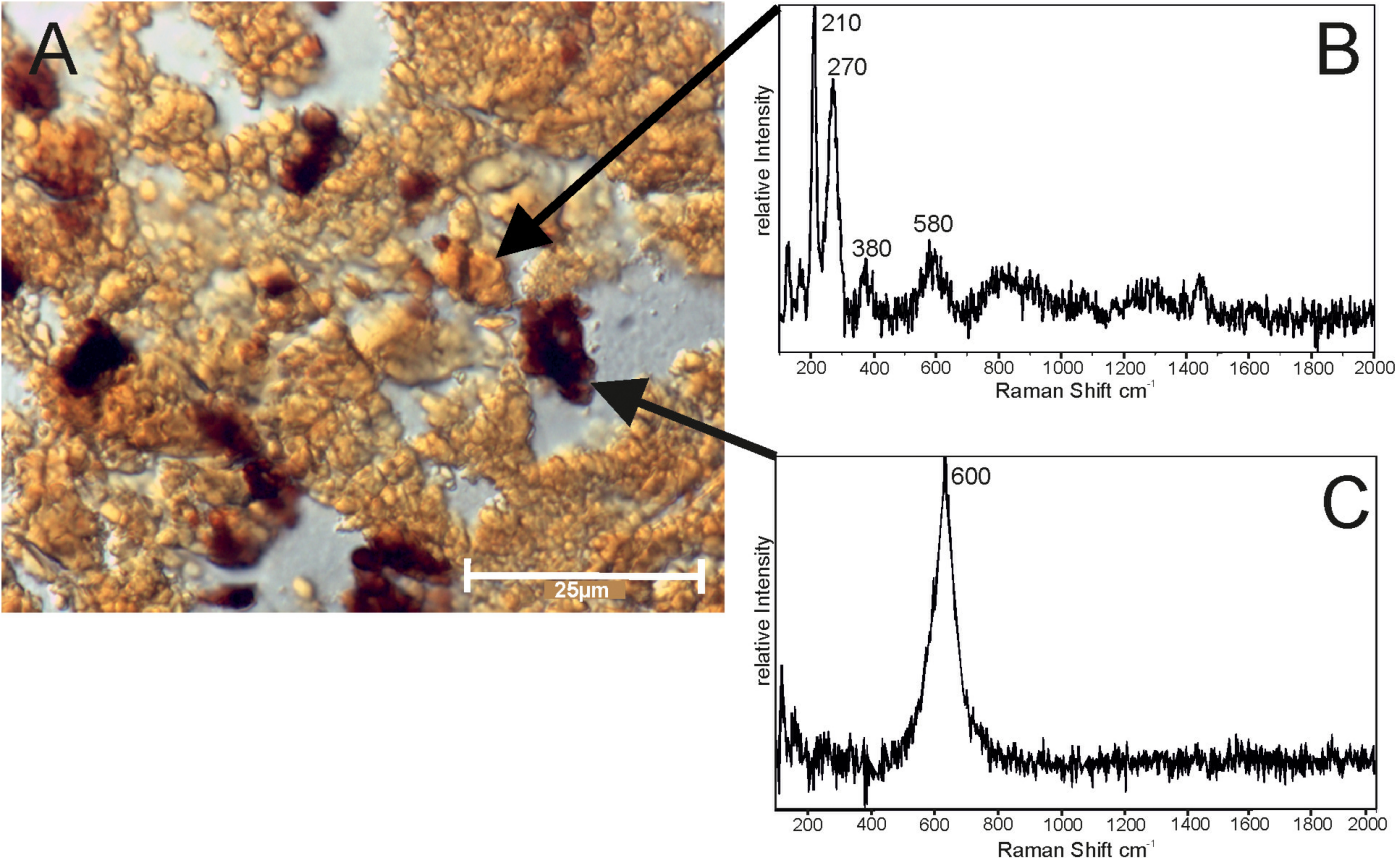
Raman spectra of two distinct areas, applied to a cross section of dendrites located in the central area of the *Frutexites*-like biofilm (A; see also 6E), exhibiting most likely hematite (B) within the red-brown, and magnetite (C) within dark aggregates. Both peaks are shifted to lower wave numbers, in close relation to organic matter peaks (O-H, C-H and C-C stretching).

Preliminary microscopic investigations indicated that the *Frutexites*-like biofilm consists of iron hydroxides and iron oxides. X-ray diffraction measurements confirmed that most of mineral precipitates are 2-line ferrihydrite (data not shown). High resolution analyses with Raman spectroscopy clearly revealed two different mineral phases, a red-brown and a dark phase (Fig 10A). The former has main bands at 210 cm^-1^ and 270 cm^-1^, with several smaller bands at around 380 cm^-1^, 580 cm^-1^ and 1290 cm^-1^ (Fig 10B). The latter has a main band at 635 cm^-1^ with a shoulder at around 600 cm^-1^ (Fig 10C). In some cases additional smaller bands appear at 215 cm^-1^, 270 cm^-1^ and 350 cm^-1^. For both mineral phases additional bands in the higher wavenumber region occur that are assignable to C-C, C-H and O-H stretching vibrations.

The band positions for both mineral phases are not clearly assignable to a single iron mineral phase, but rather indicate a mixing between several mineral phases. In the case of the red-brown mineral phase the main bands (210 cm^-1^ and 270 cm^-1^) indicate a hematite structure (α-Fe_2_O_3_), but are considerably shifted to lower wavenumbers. Normally, these bands are expected to appear around 225 cm^-1^ and 293 cm^-1^ [32, 33]. This band shifting can be explained by the influence of the laser power on the sample. This kind of laser-induced band shifting accompanied by a broadening of the band was already described by [33]. The bands of the spectra described here are even more shifted but also are much broader. Similar shifted band positions were reported by [34] for ferrihydrite which was transformed into hematite due to thermal influence of the laser excitation. During our experiments, it is likely that the laser power caused a transformation from ferrihydrite into hematite. Another explanation for these shifted band positions could be a proportion of either FeOOH (goethite) and/or FeO (wüstite) in the sample. A comparison of our spectrum with that of iron borate vonsenite (Fe_2_FeO_2_ (BO_3_; RRUFF database (RRUFF ID: R050221; [35]) shows that the bands can be related to mixed mineral phases of Fe_2_O_3_ (hematite) and FeO. The existence of the smaller broad band around 380 cm^-1^ supports the influence of FeOOH in the spectrum.

The main bands of the dark mineral phase are possibly assignable to Fe_3_O_4_ (magnetite), but are also shifted to lower wavenumbers. It is also possible that in this case a proportion of FeO (wüstite) is responsible. In the literature bands for magnetite and wüstite were reported to be the same [32], but in wüstite, the main band is shifted to lower wavenumbers, due to non-equivalent sites in the wüstite structure [33].

The elemental analysis revealed that the iron content is quite high and comprises up to 40% (406 mg/g) of the lyophilized *Frutexites*-like biofilm (Table 2). Small amounts of Ca, Si and Mn occur along with iron oxides. The organic carbon (C_org_) is rather low (4.5%). Trace amounts of inorganic carbon (C_inorg_), nitrogen (N_total_) and sulfur (S_total_) were also detected (Table 2). Carbon phase analyses indicate that the inorganic carbon is present as siderite (FeCO_3_), verified by comparison with a siderite standard.

**Table 2:** Major elemental composition of the *Frutexites*-like biofilm. The amounts are given in mg/g or respectively % dry weight.

	**Fe**	**Ca**	**Si**	**Na**	**Mn**	**Mg**	**Sr**	**Ba**	**K**
**mg/g**	406.3	29.74	28.45	5.50	14.79	4.57	0.570	0.715	0.469
** **	**C**_**total**_	**C**_**org**_	**C**_**inorg**_	**N**_**total**_	**S**_**total**_				
**%**	5.12	4.54	0.59	0.371	0.255				

All in all, the *Frutexites*-like biofilm largely consists of ferrihydrite. Minor phases of Fe(III) containing minerals are present, such as hematite and goethite (and magnetite). Small amounts of Fe(II) containing phases such as siderite and magnetite (together with wüstite) were also detected.

**Fig 11:**
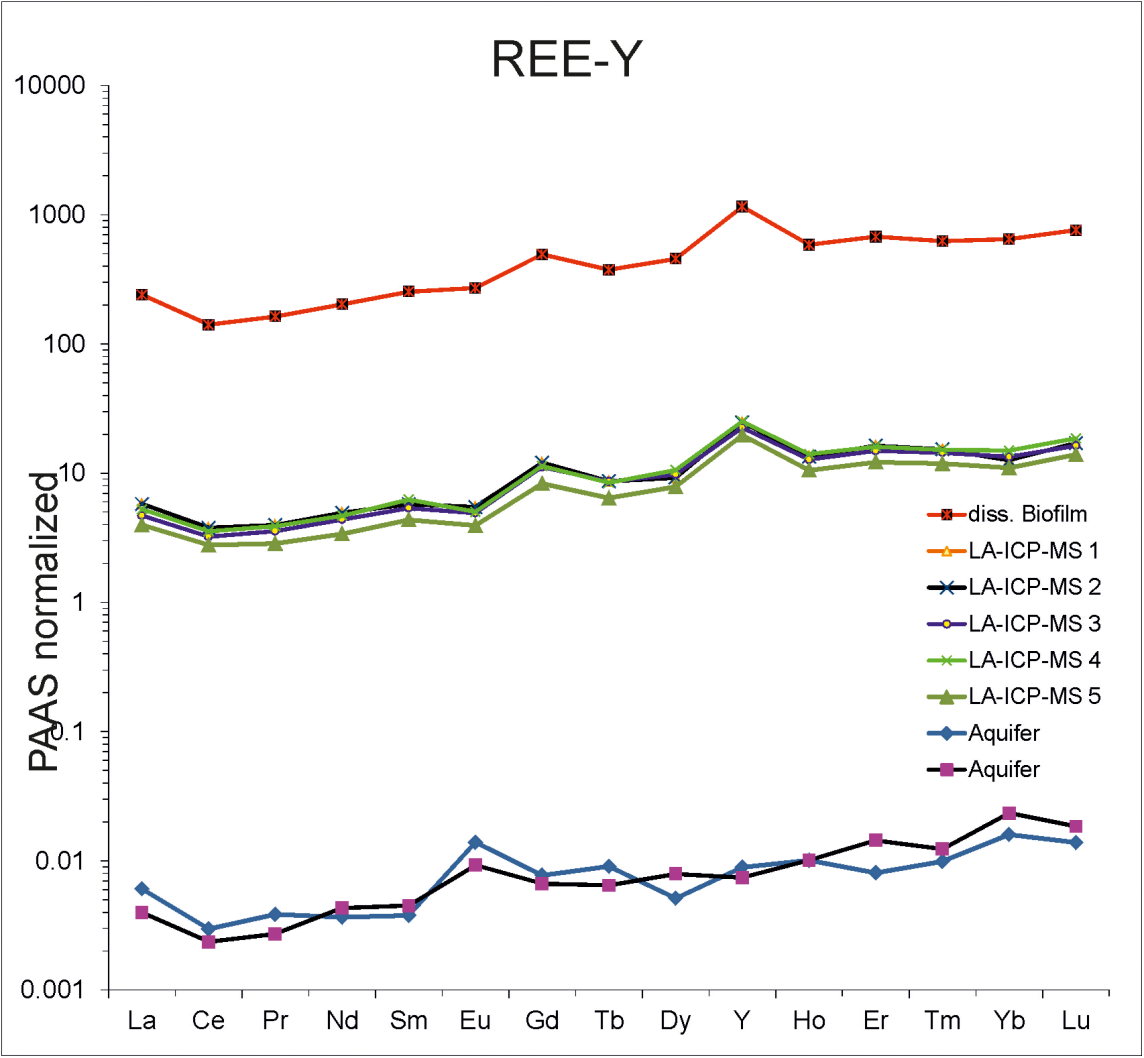
REE data from the *Frutexites*-like biofilm and the corresponding aquifer. Note that the REE-Y pattern in the biofilms follows completely the same trend as the REE-Y pattern of the aquifer.

REE-Y were analyzed to compare the REE-Y concentrations in the feeding aquifer and the dissolved biofilm (Fig 11; Table 3). The REE-Y fractionation pattern of the aquifer shows a preferential enrichment of heavy REE-Y over light REE-Y and a minor positive europium (Eu) anomaly. The REE-Y pattern of the biofilm (La-ICP-MS and dissolved biofilm) mirrors the fractionation pattern of the aquifer, except for a little positive Y anomaly. The biofilm shows an up to 10^5^-fold enrichment of REE-Y compared to the aquifer.

**Table 3:** Rare earth element and yttrium (REE-Y) concentrations of the aquifer and the *Frutexites*-like biofilm (LA-ICP-MS measurements and dissolved biofilm). The amounts are given in ng/g.

	Aquifer	Aquifer	LA-ICP-MS 1	LA-ICP-MS 2	LA-ICP-MS 3	LA-ICP-MS 4	LA-ICP-MS 5	diss. Biofilm
La	0.232	0.152	114.0	221.0	179.4	200.7	151.5	9171
Ce	0.236	0.188	177.0	301.0	256.0	282.3	220.7	11213
Pr	0.034	0.024	15.5	35.0	31.4	34.4	25.2	1441
Nd	0.124	0.146	76.0	167.0	148.3	158.8	114.7	6868
Sm	0.021	0.025	17.0	32.0	29.8	34.7	24.2	1407
Eu	0.015	0.010	3.4	5.9	5.3	5.4	4.3	293
Gd	0.036	0.031	29.6	56.1	52.0	52.9	38.7	2299
Tb	0.007	0.005	1.8	6.7	6.7	6.5	5.0	290
Dy	0.024	0.037	14.5	43.2	45.7	49.5	36.8	2133
Y	0.241	0.200	343.0	667.0	607.0	679.0	532.0	31209
Ho	0.010	0.010	4.7	13.3	12.6	13.9	10.4	580
Er	0.023	0.041	23.5	46.5	42.5	45.7	34.7	1928
Tm	0.004	0.005	2.9	6.2	5.8	6.2	4.8	253
Yb	0.045	0.066	15.4	35.5	37.7	42.2	31.0	1828
Lu	0.006	0.008	3.1	7.4	7.1	8.0	6.0	329

## Discussion

Stromatolitic Fe-oxide precipitating biofilms growing on rock surfaces at 160 m depth in the Äspö HRL were investigated. Microscopic investigations revealed that these biofilms exhibit laminar and dendritic patterns that strongly resemble those of fossil *Frutexites* structures, and are quite similar in size and shape to those originally described by Maslow [18]. A comprehensive geomicrobiological approach including FISH, 454 pyrosequencing, Raman spectroscopy, biomarker and elemental analyses allowed further elucidation of the architecture of these microbial systems.

### Microbial community

The *Frutexites*-like biofilm harbors bacteria closely associated with the known iron oxidizers *Mariprofundus ferrooxydans* and *Gallionella ferruginea*, and the iron reducer *Magnetospirillum magneticum*, which are directly involved in the iron cycle. The presence of these organisms in the Äspö HRL is well known, they have been found and analysed already in detail [24, 36, 37].

The dominant microorganisms within the recent *Frutexites* structures are relatives of ammonia oxidizing bacteria (AOB, ammonia oxidation to nitrite) and nitrite oxidizing bacteria (NOB, nitrite oxidation to nitrate). This is unexpected because nitrification provides very little energy and accordingly AOB and NOB rarely dominate microbial communities. While recent publications have identified members of the genus *Nitrospira* that are capable of performing ammonia and nitrite oxidation in conjunction, and thereby gaining more energy [38, 39], the presence of NOB suggests that this is not the case in the *Frutexites-*like structures. This is further supported by the high abundance *Candidatus Nitrotoga*, an important and efficient nitrite oxidizer, which is capable of nitrite-dependent autotrophic carbon fixation. This organism requires much lower nitrite concentrations compared to needs of *Nitrospira* and *Nitrobacter* [40]. In general, the predominance of NOB within microbial communities is often the result of a low C/N ratio [41]. This is in agreement with the C/N ratio of 12/1 for the *Frutexites*-like biofilm.

### Mineral formation within the biofilm

The iron oxidizing bacteria *Mariprofundus* sp. and *G*. *ferruginea* are known to produce massive amounts of twisted stalks made of EPS and ferrihydrite. These stalks are known to form huge amounts of microbial mats at groundwater outflows in the Äspö HRL and accumulate high amounts of trace elements [e.g. 37, 42]. In the *Frutexites* biofilms, however, stalks occur only occasionally (Fig 4A), so they are not responsible for the main structures within the biofilm. Furthermore, the presence of dominant nitrifying bacteria affects ferrous iron (Fe(II)) availability for iron oxidizing bacteria that require Fe(II) for their metabolism. Ferrous iron has long been known to accelerate the oxidation of ammonia and nitrite [43]. In contrast, microbial ferric iron (Fe(III)) reduction inhibits the reduction of nitrate or nitrite [44]. It is likely that Fe(III) formation results from indirect (i.e. not enzymatically mediated) microbial oxidation of Fe(II) by nitrite produced via bacterial respiration of nitrate [45]. Another significant aspect which influences mineralization processes are reactive organic interfaces like cell surfaces and EPS which may act as mineralizing templates [46, 47]. Functional groups on cell surfaces and in the EPS may easily connect to free cations, thereby forming complexes, which often lead to mineral nucleation. EPS in biofilms is composed of polysaccharides, proteins, lipids and humic substances, and also free nucleic acids [48, 49]. The Alcian blue stained sections (Fig 3) and the TEM sections (Fig 5) exhibit a significant amount of EPS, particularly accumulated around the cells and the tips of the dendritic structures where the mineral nucleation takes place. In general, FE-SEM, TEM and FISH analysis show a tight association of biotic and mineral structures. It has been considered that a portion of the Fe-minerals may derive from physico-chemical precipitation; however an exact differentiation of abiotic and biotic mineralization processes within recent *Frutexites* is not possible. Recent studies on biotic vs abiotic Fe mineralization in microbial mats came to a similar conclusion [50].

### REE-Y

The PAAS normalized REE-Y pattern of the aquifer is quite typical for marine waters. This is plausible, as the aquifer is highly influenced by Baltic Sea water which has the same fractionation pattern [e.g. 21, 22]. It has been shown that iron oxides, regardless of their origin (biologic or abiotic) tend to mirror the REE-Y pattern of the respective fluid [37] Therefore, the REE-Y pattern of the *Frutexites*-like biofilm only indicates the marine origin of the fluid source. This is in common with other fossil iron oxide depositions like banded iron formation [e.g. 51, 52]. An element fractionation pattern related to iron oxidizing microbial activity has not been observed so far.

### Lipid biomarkers

The most abundant saturated fatty acids (FA) C_16_ and C_18_ are unspecific, as they are common in most bacteria and eukaryotes. Although the number and position of double bonds in unsaturated fatty acids may be indicative for certain groups of recent organisms, their preservation potential is rather low. Therefore, the focus of the discussion is placed on compounds which are suitable biomarkers for the long term fossil record, like i.e. fatty acids with methyl branches, steroids or hopanoids.

Iso- and anteiso branched FA like *i-* and *ai*-C_15_; *i*-C_16_, 10Me-C_16_ and *i*-C_17_ are commonly produced by gram-positive bacteria and some anaerobic bacteria [53, 54, 55, 56, 57]. When occurring in higher amounts, iso- and anteiso branched C_15_ and C_17_ FA are often associated with sulphate reducing bacteria [e.g. 58, 59, 60, 61, 62]. The relatively low abundance of branched FA in the TOE is in a similar range as it has been described for different NOB [31].

The organic compounds diploptene, diplopterol were reported to be highly abundant in *Nitrosomonas europaea* [63]. This organism also produces high concentration of aminohopanetriol, but this compound was not observed. Other hopanepolyols were detected in considerable amounts, but they are common in many bacteria. The branched hydoxy fatty acid trihydroxy-*i*-C_11_, and trihydroxy-*i*-C_17_ which are abundant in *Mariprofundus* sp. [64] were not detected. Notably, the *Frutexites*-like biofilm contained numerous sterols, indicating a significant eukaryotic contribution to the biofilm. Cholesterol, sitosterol, ergosta-5,22-dienol and ergosta-5,7-dienol have been found in several micro algae [65, 66, 67, 68]. Even though the most typical fungal sterol (ergosterol) is lacking, most of the sterols observed in the *Frutexites*-like biofilm could also derive from fungi [e.g. 69]. As both algae and fungi can form net-like structures and dendritic patterns; their contribution to the “typical *Frutexites* structure” is plausible. A symbiotic relationship of bacteria and fungi leading to the formation of *Frutexites* has recently been hypothesized [70].

Despite the observation of archaea with FISH (Fig 7D and 7H), characteristic isoprenoid biomarkers like pentamethylicosane, archaeol and or hydroxyarchaeol have not been detected. Archaeal tetraether lipids may be present, but have not been analyzed. This initial chemical investigation of the *Frutexites*-like biofilm implies that the diversity of this biofilm is quite complex and requires more in-depth studies to determine the interaction of the different prokaryotes and eukaryotes that form these structures.

## Conclusion

Our results make a strong case for the contributions of prokaryotic and eukaryotic organisms to the formation of *Frutexites*-like structures. The recent *Frutexites*-like biofilm contained numerous archaeal and bacterial microorganisms. Lipid biomarker analysis indicated the presence of eukaryotes, namely algae and fungi. The identification and functional role of algae and/or fungi and their contribution to the dendritic structure of *Frutexite*s need to be investigated further. Furthermore, the detected biomarkers are suitable for comparable biomarker studies in ancient *Frutexites* in the future. Side-by-side occurrences of Fe(II) and Fe(III) mineral phases can be used as an indicator for biologically governed microenvironments. The association of such mineral phases may also be a “signature” to look for in fossil environments.
